# Sample preparation conditions for the real-time measurement of W/O emulsions by resonance-enhanced multiphoton ionization time-of-flight mass spectrometry

**DOI:** 10.1007/s44211-023-00486-3

**Published:** 2024-01-09

**Authors:** Minori Minami, Shion Nakata, Tomohiro Uchimura

**Affiliations:** Department of Materials Science and Engineering, Graduate School of Engineering, University of Fukui, 3-9-1 Bunkyo, Fukui, 910-8507 Japan

**Keywords:** W/O emulsion, REMPI, TOFMS, Real-time measurement

## Abstract

Analysis of an emulsion in its original dispersed condition is quite important for quality assessment and quality control. In the present study, the practical experimental conditions of the real-time measurement of a water-in-oil (W/O) emulsion were examined via resonance-enhanced multiphoton ionization time-of-flight mass spectrometry (REMPI-TOFMS). A W/O emulsion was prepared using cyclohexane as the oil phase with toluene as an analyte species. A time profile of the peak area for toluene was constructed based on the mass spectra. Normally, the negative spikes of a base signal are detected in a time profile when analyte molecules are dispersed in an oil phase. In this case, however, the positive spikes were unexpectedly detected rather than the negative ones. Though several factors could be relevant for the occurrence of the positive spikes, these spikes could have been suppressed by the addition of a small amount of *n*-alkane when the oil phase was prepared in the present study. The practical experimental conditions for the analysis of a W/O emulsion in real-time revealed that this method would be applicable to the analysis of an oil-in-water-in-oil (O/W/O) emulsion where the outer phase is also an oil phase.

**Graphical abstract:**

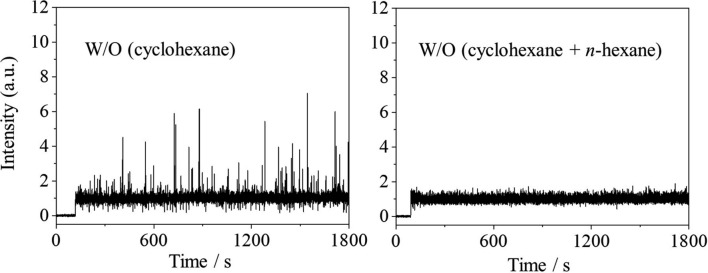

**Supplementary Information:**

The online version contains supplementary material available at 10.1007/s44211-023-00486-3.

## Introduction

An emulsion is a mixture of two (or more) liquids that are immiscible, one of which is dispersed in the form of small droplets throughout the other. Simple emulsions have structures of either oil-in-water (O/W) or water-in-oil (W/O). Oil-in-water-in-oil (O/W/O) and water-in-oil-in-water (W/O/W) emulsions are referred to as multiple (double) emulsions. Emulsions are evaluated via several analytical methods, the simplest of which is to assess stability using appearance and turbidity [1, 2]. The measurement of conductivity or backscattering light is used to evaluate phase inversion according to temperature change [3, 4]. Mass spectrometry (MS) is a powerful tool that is used to identify the analyte species according to the values of the mass-to-charge ratio (*m*/*z*). Historically, droplets formed by passing through a microchip have been measured via electrospray ionization quadrupole mass spectrometry (ESI-QMS) [5–7].

For the quality control and quality evaluation of an emulsion, it is quite important to analyze the constituents existing in each phase of the original dispersed condition. We have demonstrated the direct analysis of an emulsion without pretreatment by resonance-enhanced multiphoton ionization time-of-flight mass spectrometry (REMPI-TOFMS) [8]. This method is applied mainly to the measurement of gaseous samples [9–12]. By contrast, we proposed a sample introduction technique for measuring a liquid sample [8], and a brief explanation follows. First, we found that the sample was explosively introduced when a single capillary column was used for the sample introduction. To smoothly introduce the sample, a pair of concentric capillary columns was constructed, so that a liquid sample passes through an inner capillary column after ambient air is introduced from an outer column. When ultraviolet laser pulses are utilized for ionization, in many cases, aromatic compounds with the same absorption wavelength as that of the laser wavelength are selectively ionized and detected. In an emulsion measurement, when an aromatic compound is used as an analyte species, such a hydrophobic compound should distribute in an oil phase. By continuously introducing an emulsion into a TOFMS, a series of mass spectra are obtained, and time profiles of the peak areas of the analyte species are successively obtained. When an O/W emulsion is measured, a weak base signal with positive spikes appears on the time profiles. Positive spikes indicate that the analyte molecules are localized in an O/W emulsion, which verifies the presence of oil droplets. We have reported that toluene droplets with diameters of ca. 3 µm or more are detectable as positive spikes when an inner capillary column with an inner diameter of 25 µm is used for the sample introduction [13]. The appearance of a weak base signal is attributed to a large number of quite small oil droplets and to the slight distribution of hydrophobic analytes in the water phase.

In the case of a W/O emulsion measurement where the outer phase is an oil phase, a base signal should arise from the oil phase and negative spikes should be detected from the water droplets, and such time profiles were actually obtained [14]. However, as will be discussed subsequently, positive spikes were dominantly detected when measuring a W/O emulsion; such results were not occasional, but were in fact reproducible. Positive spikes suggest an instantaneous increase in the amount of the analyte species in the ionization region, which perhaps happens, because a W/O emulsion is explosively introduced into a TOFMS. In the present study, we confirmed the reproducibility of the unexpected positive spikes, and verified that the experimental conditions needed to suppress the positive spikes involved changing the ratios of the oil components.

## Experimental

### Reagent and preparation of samples

Cyclohexane, *n*-pentane, *n*-hexane, *n*-nonane, toluene, and sorbitan monooleate (Span 80, as an emulsifier) all were purchased from FUJIFILM Wako Pure Chemical Corporation (Osaka, Japan) and were used without further purification.

The preparation procedures for a W/O emulsion were as follows. First, solutions for an oil phase were prepared; the ratios of cyclohexane and one of the *n*-alkanes were adjusted to 100:0, 99.7:0.3, 99:1, and 90:10 (v:v), respectively. Then, toluene was added, and the concentration was adjusted to 5 g/L (i.e., 0.125 g of toluene per 25 mL of the solution for an oil phase). Also, 0.258 g of Span 80 was added to another vial container, and 25 mL of the solution for an oil phase were added. While stirring the solution with a homogenizer (4000 rpm), 0.773 mL of distilled water was added using a micropipette. The ratio between distilled water and the solution for the oil phase was 3:97 (v:v). The stirring was continued for 3 min, and a W/O emulsion was obtained. The final concentration of Span 80 was 10 g/L of a W/O emulsion.

### REMPI-TOFMS

Details of the REMPI-TOFMS used in the present study are reported elsewhere [8]. Briefly, a pair of concentric fused-silica capillary columns was used for the sample introduction. The inner/outer diameters and the length of the inner capillary column were 50/150 µm and 115 cm, respectively, and those of the outer capillary column were 320/450 µm and 25 cm, respectively (GL Sciences, Tokyo). In the present study, the inner diameter of the inner capillary column was larger than that used in the previous studies (25 µm) [14, 15]. In future studies, the use of an inner capillary with a larger inner diameter should allow an emulsion containing larger droplets (e.g., water droplets in the case of a W/O emulsion or O_1_/W droplets in the case of an O_1_/W/O_2_ emulsion) to smoothly pass. Ambient air was introduced from an outer capillary column; a flow meter was used to adjust the flow rate to 2 mL/min. During the measurement, the sample was gently stirred with a magnetic stirrer (300 rpm) to suppress the creaming of the W/O emulsion.

The fourth harmonic generation of a pulsed Nd:YAG laser (Rayture Systems, GAIA II, 266 nm, 4 ns, 10 Hz, Tokyo) was used for REMPI, and toluene molecules could be selectively ionized among the components in the W/O emulsion. The laser pulses were focused using a lens (*f* = 200 mm) and were aligned 2 mm away from the tip of the outer capillary column. A sample was measured for 30 min; the recording was started with the simultaneous insertion of the inner capillary column into a sample. The peak areas for toluene, which included the molecular ion peak (*m*/*z* 92) and the fragment ion peak (*m*/*z* 91), were extracted from the mass spectrum, and a time profile of the peak area for toluene was constructed using the series of the mass spectra. Each individual signal intensity of the time profile was divided by the average signal intensity (calculated from the data obtained for 3–30 min) to achieve normalization. For daily system evaluation, a toluene solution (in cyclohexane) was measured at the beginning of each measurement day, and we confirmed that no spikes were detected on the time profile, which meant that an explosive introduction of the sample was avoided to allow a smooth introduction into the TOFMS.

### Optical microscope

Using an aspirator, a W/O emulsion was aspirated through a capillary column with the same inner/outer diameters as the inner capillary column. The conditions of the aspirated sample passing through the capillary column were observed using an inverted optical microscope (ECLIPSE TE2000-U, Nikon, Tokyo) with a 20 × objective (Plan Fluor, numerical aperture 0.45, Nikon).

## Results and discussion

**Fig. 1:**
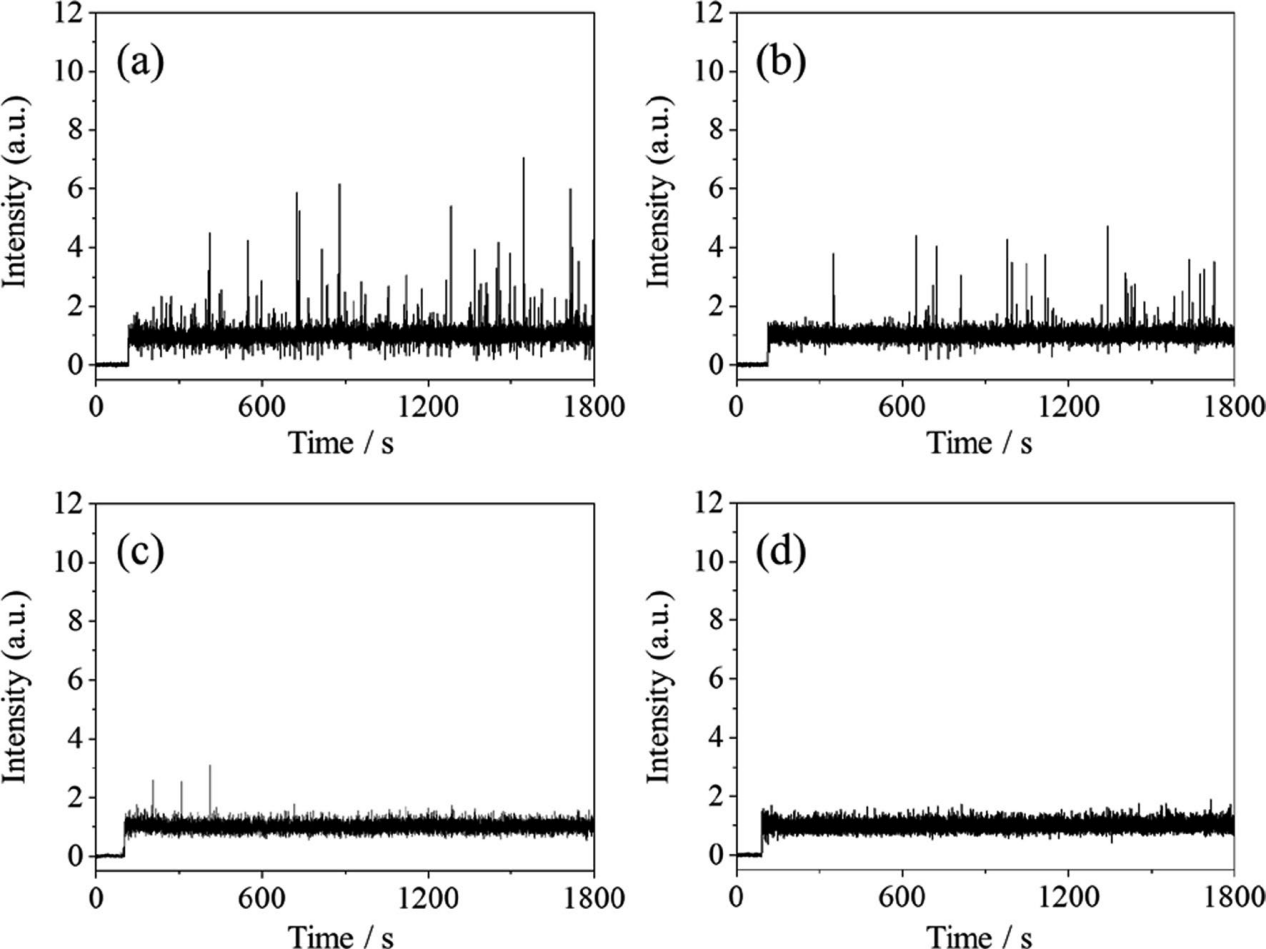
Time profiles of the peak areas for toluene in W/O emulsions. Oil phase: cyclohexane and *n*-hexane. Ratios of cyclohexane and *n*-hexane (v:v): 100:0 (**a**); 99.7:0.3 (**b**); 99:1 (**c**); and, 90:10 (**d**)

The obtained time profiles of the peak areas for toluene in the W/O emulsions are shown in Fig. 1. Signals were detected ca. 100 s after starting the recording; this indicates the time needed for the passage of the emulsion through an inner capillary column. The linear velocity was calculated to be roughly 1 cm/s based on the time and the length of the inner capillary (115 cm).

As mentioned previously, first, we assumed that negative spikes based on the base signal would appear on the time profile when a W/O emulsion containing toluene as an analyte species was measured. However, as shown in Fig. 1a where cyclohexane was only used for the oil phase, positive spikes, rather than negative spikes, were remarkably detected.

**Fig. 2:**
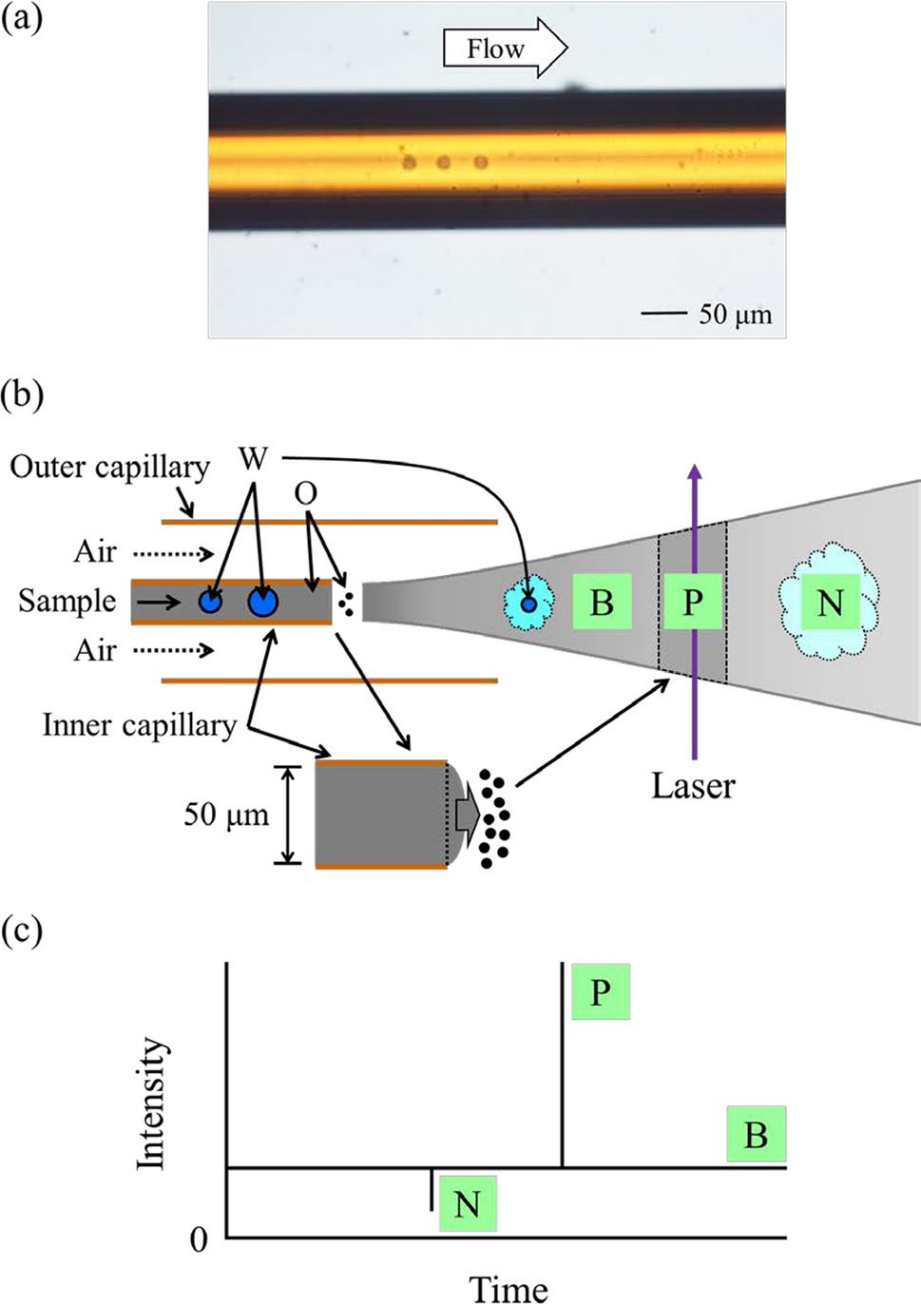
Schematic illustration depicting the mechanism of the generation of the base (B), negative (N), and positive (P) signals on a time profile. Microscopic image of a W/O emulsion flowing through a capillary column. Ratios of cyclohexane and *n*-hexane: 100:0 (v:v) (**a**); schematic illustration of a W/O emulsion introduced into a TOFMS (**b**); schematic illustration of a time profile (**c**)

Figure 2 shows a schematic illustration depicting the mechanism for the generation of the base (B), negative (N), and positive (P) signals on the time profile. Although the condition of an emulsion after introduction into a TOFMS is unknown, conventional wisdom would predict that small oil droplets generated by nebulizing the oil phase would eventually become quite small droplets (or vaporized molecules) when reaching the ionization region. As a result, a base signal is obtained in the time profile.

Water droplets in a W/O emulsion are also considered to be gradually vaporized after introduction into a TOFMS. By nebulizing a large water droplet, a larger region existing in toluene is formed in quite small concentrations, compared with the region where the laser beam passes, which results in the emergence of a negative spike. Herein, we calculated the size of a water droplet flowing through an inner capillary column would be needed for the appearance of a negative spike, which is based on the absolute value of the signal intensity of a negative spike (*S*) and on the fluctuation of the base signal (*F*). In the present study, the latter value was determined to be twice the standard deviation of the signal intensities of the base signal, which was obtained by the time profile when measuring a toluene solution (data not shown). By referring to the equation described in our previous report [13], the minimum size of a detectable droplet that would produce a negative spike would be 48 µm, by assuming that a spike with an *S*/*F* = 3 would be detectable. That is to say, under the present experimental conditions, the sensitivity for the detection of the decrease in the amount of toluene was low, and a negative spike was only detectable when the size of a water droplet was roughly equivalent to the inner diameter of the inner capillary column.

By contrast, the appearance of positive spikes seemed to exist for the toluene locally in the W/O emulsion. This was difficult to imagine, because it is improbable that the concentration of toluene in a water phase would be higher than that in the oil phase. If toluene droplets were formed inside a water droplet, that is, if an O_1_(toluene)/W/O_2_(cyclohexane) emulsion were obtained and were measured, the positive spikes would appear. However, special methods that include a two-step emulsification are needed to prepare such a multiple emulsion [15]. It should be impossible to obtain such emulsions under the present preparation conditions, and such emulsions were not confirmed by observation via an optical microscope. Hence, we considered that the appearance of positive spikes did not indicate the localization of toluene in the W/O emulsion measurement. Instead, we assumed that the positive spikes had derived from a slight increase in the volume of the emulsion via the introduction into TOFMS due to the instantaneous and unexpected explosive manner of the introduction (Fig. 2). In the present study, an inner capillary column with an inner diameter of 50 µm was applied, instead of that of 25 µm that was used in the previous studies [14, 15]. Due to the use of the inner capillary column with a larger inner diameter, we had expected such an explosive sample introduction; as a countermeasure, a toluene solution (in cyclohexane) had been measured at the beginning of each measurement day and no spikes were detected on the time profile. Based on these results, we concluded that the volume of the sample introduction must have occasionally increased when introducing the W/O emulsion prepared in the present study. This could have been the case, because the sample stayed slightly on the tip of the inner capillary column, while no explosive sample introduction occurred when introducing the toluene solution. Incidentally, although the amount of toluene is considered to be decreased before and/or after the occurrence of the explosive sample introduction, the decrease in the signal intensity was confirmed neither before nor after the positive spikes in the time profile shown in Fig. 1a. This is probably due to the low sensitivity for the detection of the decrease in the amount of toluene, as mentioned previously.

The occurrences of positive spikes are irrelevant for the localization of an analyte species (toluene) in a W/O emulsion, and this is undesirable for the direct analysis of a W/O emulsion in real time. Hence, the experimental conditions that caused (and would suppress) the appearance of positive spikes were verified. In the present study, several W/O emulsions with different ratios of cyclohexane and *n*-hexane [99.7:0.3, 99:1, and 90:10 (v:v)] were monitored (Fig. 1b–d) to check the relationship between the appearance of positive spikes and the composition of the oil phase that accounts for a large fraction of a W/O emulsion. As a result, the positive spikes of toluene decreased as the ratio of *n*-hexane increased among the prepared W/O emulsions. Figures S1 and S2 show the time profiles when measuring W/O emulsions using the mixture of cyclohexane and other *n*-alkanes, namely *n*-pentane and *n*-nonane, as an oil phase. Similar to the results obtained when using *n*-hexane, the positive spikes of toluene decreased with an increase in the ratio of each *n*-alkane. Factors such as the surface tension, viscosity, and the flow rate of the sample, in addition to the inner diameter of the inner capillary column for sample introduction, are considered to be relevant for an instantaneous and slight increase in the volume when a sample is introduced. The surface tension and the viscosity of the compounds used as the oil phase are shown in Table S1. Both values of the *n*-alkanes used in the present study are smaller than those of cyclohexane, and, therefore, both values are considered to be decreased by mixing cyclohexane and one of the *n*-alkanes compared with when only cyclohexane is used. Although it is unclear how much these values affected the fluctuation of the volume of the sample introduction, in any case, the positive spikes had disappeared following the addition of a small amount of *n*-alkane to the W/O emulsion used in the present study, which is practical for the measurement of a W/O emulsion in real time.

The results of the present study demonstrate that a W/O emulsion could be directly measured in real time using REMPI-TOFMS. In future studies, this method will be applied to the measurement of an O_1_/W/O_2_ emulsion, the outer phase of which is also an oil phase. When inner oil droplets include a detectable component, positive spikes arising from that component should be obtained. Such studies verify the kinetics of the release of a component from an inner to an outer oil phase. Though only toluene was used as an analyte in the present study, REMPI-TOFMS can, of course, simultaneously and separately detect compounds with different *m*/*z* values. Therefore, the moving behavior of multiple oil components between the oil phases in an O/W/O emulsion could be directly evaluated.

### Supplementary Information

Below is the link to the electronic supplementary material.

Supplementary file1 The time profiles of the peak areas for toluene in W/O emulsions (oil phase: cyclohexane and n-pentane or n-nonane) and the surface tension and the viscosity of the compounds used as the oil phase. (PDF 522 kb)

## Data Availability

The datasets generated during and/or analyzed during the current study are available from the corresponding author upon reasonable request.
