# Supplementary material for: Sample preparation conditions for the real-time measurement of W/O emulsions by resonance-enhanced multiphoton ionization time-of-flight mass spectrometry
Source: Anal Sci. 2024 Jan 9;40(3):573–7. doi: 10.1007/s44211-023-00486-3 (PMC10894760; doi:10.1007/s44211-023-00486-3)
Supplement: Supplementary file 1 — Supplementary file1 The time profiles of the peak areas for toluene in W/O emulsions (oil phase: cyclohexane and n-pentane or n-nonane) and the surface tension and the viscosity of the compounds used as the oil phase. (PDF 522 kb) [file 44211_2023_486_MOESM1_ESM.pdf]

Note

Sample preparation conditions for the real-time measurement of W/O emulsions by resonance-enhanced multiphoton ionization time-of-flight mass spectrometry

<S*upporting Information*>

Minori MINAMI, Shion NAKATA, and Tomohiro UCHIMURA†

Department of Materials Science and Engineering, Graduate School of Engineering, University of Fukui, 3-9-1 Bunkyo, Fukui 910-8507, Japan

† To whom correspondence should be addressed.

E-mail: uchimura@u-fukui.ac.jp

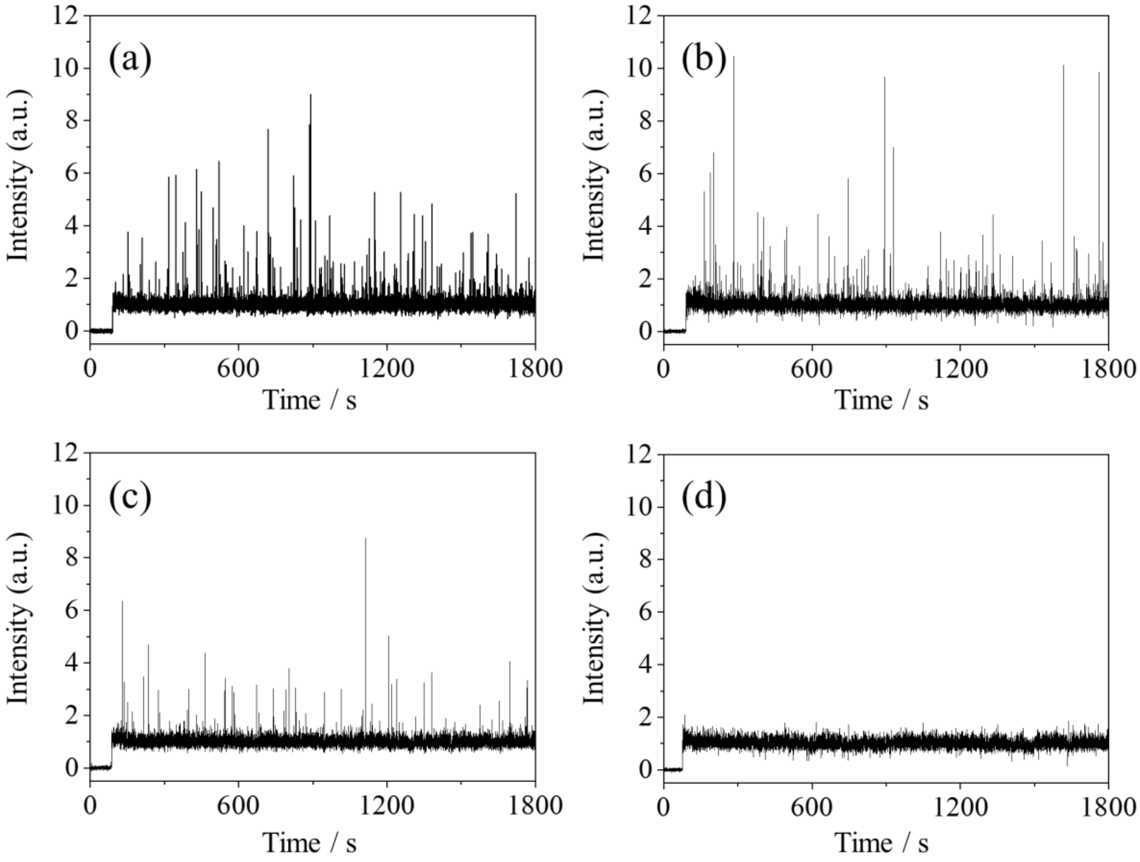

Fig. S1 Time profiles of the peak areas for toluene in W/O emulsions. Oil phase: cyclohexane and *n*-pentane. Ratios of cyclohexane and *n*-pentane (v:v): 100:0 (a); 99.7:0.3 (b); 99:1 (c); and, 90:10 (d).

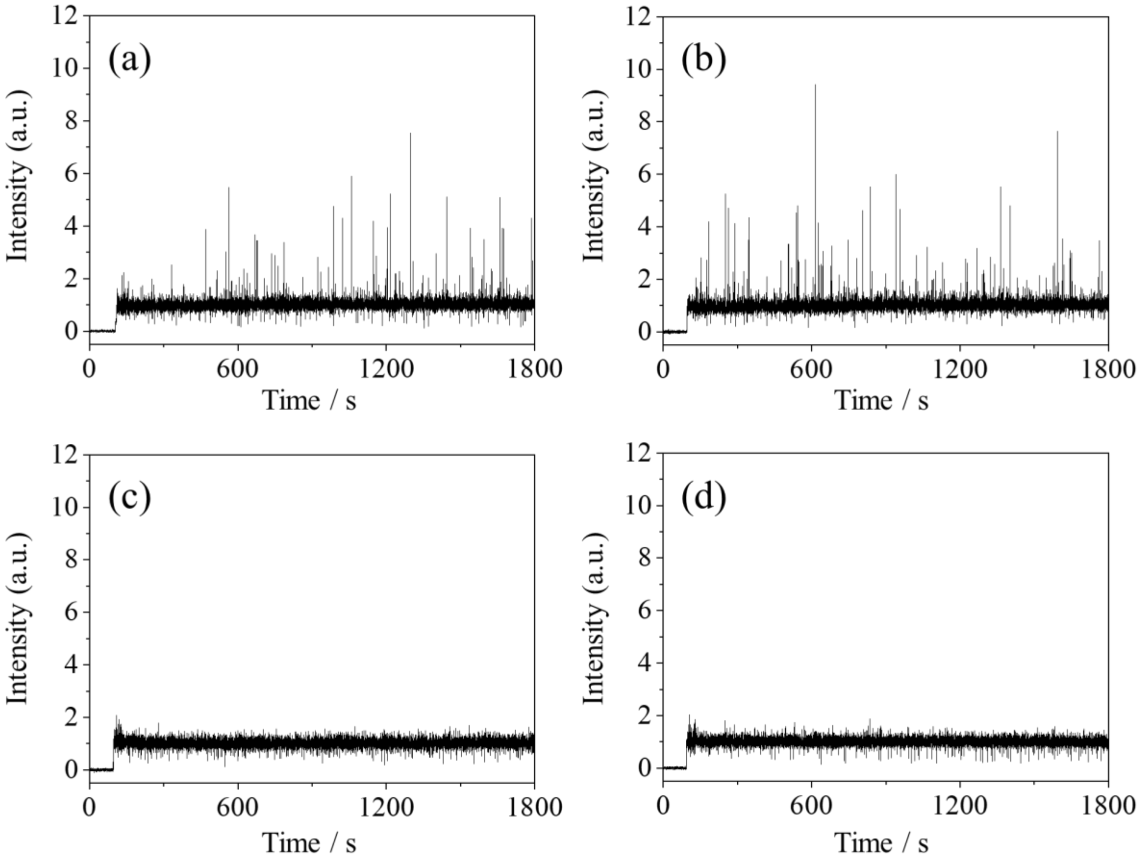

Fig. S2 Time profiles of the peak areas for toluene in W/O emulsions. Oil phase: cyclohexane and *n*-nonane. Ratios of cyclohexane and *n*-nonane (v:v): 100:0 (a); 99.7:0.3 (b); 99:1 (c); and, 90:10 (d).

]

Table S1 Surface tension and viscosity of the compounds used as an oil phase[S1]

Compound	Surface tension[a]/mN m^{-1}	Viscosity[a]/mPa s
Cyclohexane	24.65	0.894
n -Pentane	15.49	0.224
n -Hexane	17.89	0.300
n -Nonane	22.38	0.665

[a] Temperature: 25 °C

[S1] D. R. Lide, Ed., *CRC Handbook of Chemistry and Physics*, 84th ed.; CRC Press: 2003-2004.
